# Analysis of a radiation-induced dwarf mutant of a warm-season turf grass reveals potential mechanisms involved in the dwarfing mutant

**DOI:** 10.1038/s41598-020-75421-x

**Published:** 2020-11-03

**Authors:** Tianyi Lin, Ren Zhou, Bo Bi, Liangyuan Song, Mingliang Chai, Qiaomei Wang, Guoqing Song

**Affiliations:** 1 Department of Horticulture, College of Agriculture and Biotechnology, Zhejiang University, Hangzhou, 310058 China; 2 Plant Biotechnology Resource and Outreach Center, Department of Horticulture, Michigan State University, East Lansing, MI 48824 USA

**Keywords:** Biotechnology, Genetics, Plant sciences

## Abstract

*Zoysia matrella* [L.] Merr. is a widely cultivated warm-season turf grass in subtropical and tropical areas. Dwarf varieties of *Z. matrella* are attractive to growers because they often reduce lawn mowing frequencies. In this study, we describe a dwarf mutant of *Z. matrella* induced from the ^60^Co-γ-irradiated calluses. We conducted morphological test and physiological, biochemical and transcriptional analyses to reveal the dwarfing mechanism in the mutant*.* Phenotypically, the dwarf mutant showed shorter stems, wider leaves, lower canopy height, and a darker green color than the wild type (WT) control under the greenhouse conditions. Physiologically, we found that the phenotypic changes of the dwarf mutant were associated with the physiological responses in catalase, guaiacol peroxidase, superoxide dismutase, soluble protein, lignin, chlorophyll, and electric conductivity. Of the four endogenous hormones measured in leaves, both indole-3-acetic acid and abscisic acid contents were decreased in the mutant, whereas the contents of gibberellin and brassinosteroid showed no difference between the mutant and the WT control. A transcriptomic comparison between the dwarf mutant and the WT leaves revealed 360 differentially-expressed genes (DEGs), including 62 up-regulated and 298 down-regulated unigenes. The major DEGs related to auxin transportation (e.g., *PIN-FORMED1*) and cell wall development (i.e., *CELLULOSE SYNTHASE1*) and expansin homologous genes were all down-regulated, indicating their potential contribution to the phenotypic changes observed in the dwarf mutant. Overall, the results provide information to facilitate a better understanding of the dwarfing mechanism in grasses at physiological and transcript levels. In addition, the results suggest that manipulation of auxin biosynthetic pathway genes can be an effective approach for dwarfing breeding of turf grasses.

## Introduction

*Zoysia matrella* [L.] Merr., also known as Manila grass, is a perennial herbaceous plant of the Gramineae family^1^. *Z. matrella* is widely used in subtropical and tropical areas as a warm-season turf grass because of its fine-texture, the formation of dense lawn, fast spread, wearing tolerance, shade tolerance, and low nutrient requirements^2^. *Z. matrella* is mainly vegetatively propagated due to the lack of fertile seeds in nature. For breeding, *Zoysia* grasses display facultative reproduction, pistil precocious, and interspecific hybridization, which consequently lead to the prevalence and rich genetic variations in natural interspecific hybrids. This makes it difficult for traditional breeding due to the difficulty in identifying the species morphologically^3^. Therefore, new biotechnological tools such as somaclonal variation breeding and genetic engineering are more preferable to traditional breeding approaches through crossing and seed selection for *Z. matrella* breeding.

Somaclonal variation induced by callus regeneration is a major source of variation in plant breeding^4^. Artificial plant reproduction using somaclonal variation techniques drives minimum biosafety concerns and thus has become an effective way to improve turf grass in recent years. To date, effective regeneration systems in cool-season turf grasses have been reported for different species using different explants and culture conditions^5–11^. In contrast, efficient regeneration systems for warm-season turf grass are still lacking because warm-season grasses are usually not amenable for in vitro culture^12^. Thus, fewer studies on somaclonal variations in warm-season turf grasses have been reported when compared with cool-season turf grasses. Although some evidence of plant regeneration has been reported^13–16^, *Zoysia* grasses are still considered recalcitrant to produce fast-growing embryogenic callus with high regeneration ability^13,14,17^. In *Z. matrella*, it is difficult to induce embryogenic callus since the most of *Z. matrella* does not have viable seeds. The stolons and rhizomes with buds become the only explants. To enable somaclonal variation breeding and genetic transformation for *Z. matrella*, we have developed an efficient protocol of callus induction, embryogenic callus formation and long-term maintenance of embryogenic cultures and green plant regeneration for *Z. matrella* which laid a foundation for this study^18^.

Dwarfism is an important agronomic trait for a high crop yield and has been widely studied in model plants and field crops^19–21^. High-yielding dwarf wheat varieties were a major driver of the Green Revolution^22^. Genetically, many factors are involved in plant dwarfism, such as transcription factors (TFs), microRNAs (miRNAs), the plant skeletal system and cell wall related genes^23–27^. In addition, genetic variations on synthesis or signal transduction pathways of several phytohormones [i.e., including gibberellins (GA), brassinosteroids (BRs), and auxin (indole acetic acid, IAA)] also have dramatic effects on plant height and branching^28–31^. To date, many genes participating in GA biosynthesis (i.e., *TPSs, CPS, KS, P450s, KO, KAO, 2ODDs, GA20ox, GA3ox, GA2ox*), metabolism (i.e., *GA2ox, EUI, GAMT*) and signaling transduction (i.e., *GID1, DWARF1, PICKLE1, GAMYB, SLEEPY1, PHOR1, RGA, GAI, SPY, SHI, PIFs*) have been isolated and identified which were considered influencing plant height^32–38^. BR dwarf mutants (i.e., *dwfl*, *cpd/dwf3*, *dwf4*, *dwf5*, *det2/dwf6*, *stel/dwf7*, *brd1*, *det2*, *rot3, Ika, Ikb*) have also been isolated and studied^39–41^. The variation of genes in IAA pathway (i.e., *TAA1*, *YUC*, *FMO*, *P450s*, *ABCB1/PGP1*, *ARF*, *AUX/IAA*) caused plant dwarfing as well^42,43^. For turf grasses, dwarfism is desirable because it facilitates high density planting, low frequency of mowing, and high photosynthetic efficiency^44^. However, the molecular mechanism of dwarfing turf grasses has not been reported.

Radiation mutagenesis induces mutations in cells through radiation (microwave or laser radiation) and produces corresponding genetic variations^45^. Gamma rays are the most commonly used source of radiation mutagenesis^46^. In this study, gamma rays were used to induce mutations from *Z. matrella* calluses for modifying morphological traits, and a dwarf mutant was identified. We conducted morphological, physiological and biochemical, and transcriptomic characterization of the mutant to uncover possible dwarfing mechanisms in the mutant.

## Results

### Morphological characteristics of the dwarf mutant

**Figure 1:**
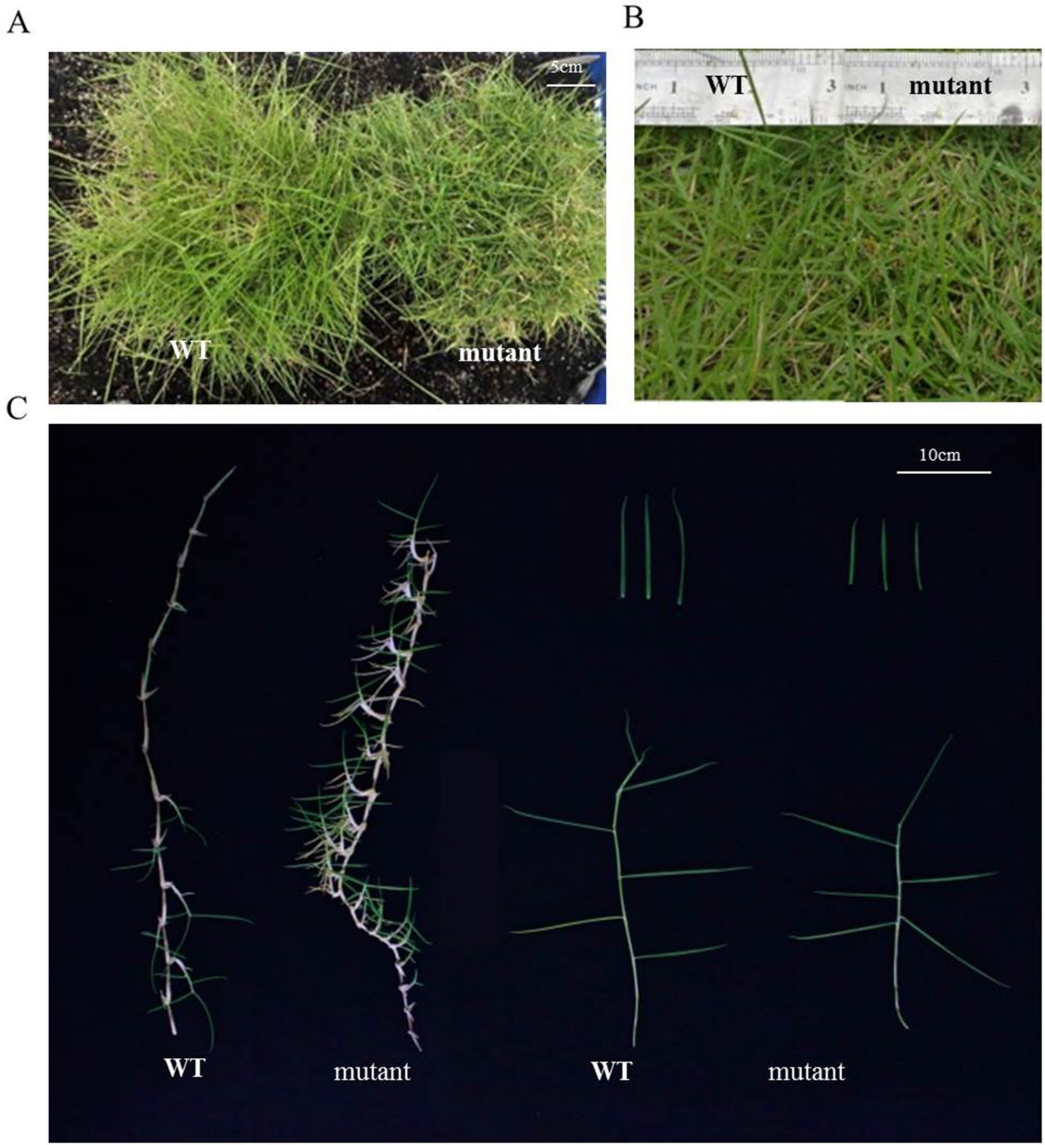
Morphological comparisons between the dwarf mutant and WT plants of *Z. matrella.* (**A**) The dwarf mutant and WT plants growing in the greenhouse for 3 years. (**B**) Leaves. (**C**) Stolons and leaves.

**Figure 2:**
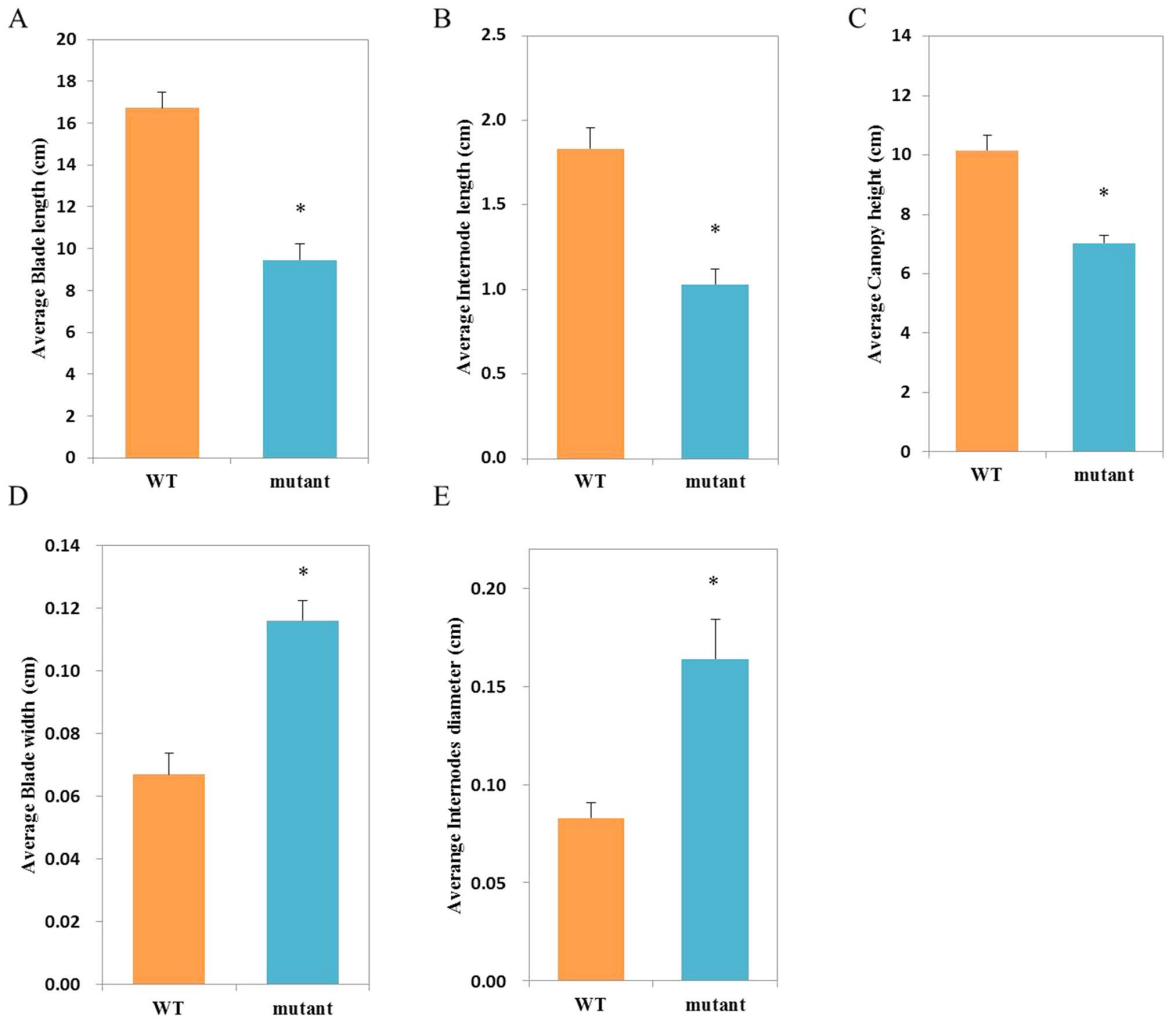
Changes in morphological indicators between the dwarf mutant and WT plants of *Z. matrella.* (**A**) Blade length. (**B**) Internode length. (**C**) Canopy height. (**D**) Blade width. (**E**) Internodes diameter. ‘*’ on error bars mean the significant differences at *P*  <  0.05 by Duncan’s multiple range tests.

When growing under the greenhouse conditions for 3 years, the mutant plants had shorter stems, wider leaves, lower canopy height and a deeper green color than the WT plants (Fig. 1A–C). The average plant height of the mutant was 69% shorter than the WT plants and showed 44% of reduction of blade length and internode length (Table 1, Fig. 2A–E). These phenotypic data confirm the dwarf characteristics in the mutant and suggest that the mutant carries stable mutation(s) for dwarfing. In addition, the anatomical structures of the mutant and WT were consistent with the results of morphological characteristics. In the cross section of leaves, the mutants had longer blade width and thickness than WT (Table 1, Supplementary Fig. 1 and Supplementary Fig. 2).

**Table 1:** Phenotypic comparison of the dwarf mutant and the WT plants of *Z. matrella*, n = 20.

Plant	Blade length (cm)	Blade width (cm)	Internode length (cm)	Internodes diameter (cm)	Canopy height (cm)
WT	16.73 ± 1.70a	0.07 ± 0.05b	1.83 ± 0.07a	0.08 ± 0.03b	10.15 ± 0.90a
Mutant	9.45 ± 1.50b	0.12 ± 0.03a	1.03 ± 0.09b	0.16 ± 0.05a	7.03 ± 1.10b

Different letters indicate statistical difference at 0.05 level according to Duncan’s tests.

### Physiological changes in the dwarf mutant

**Figure 3:**
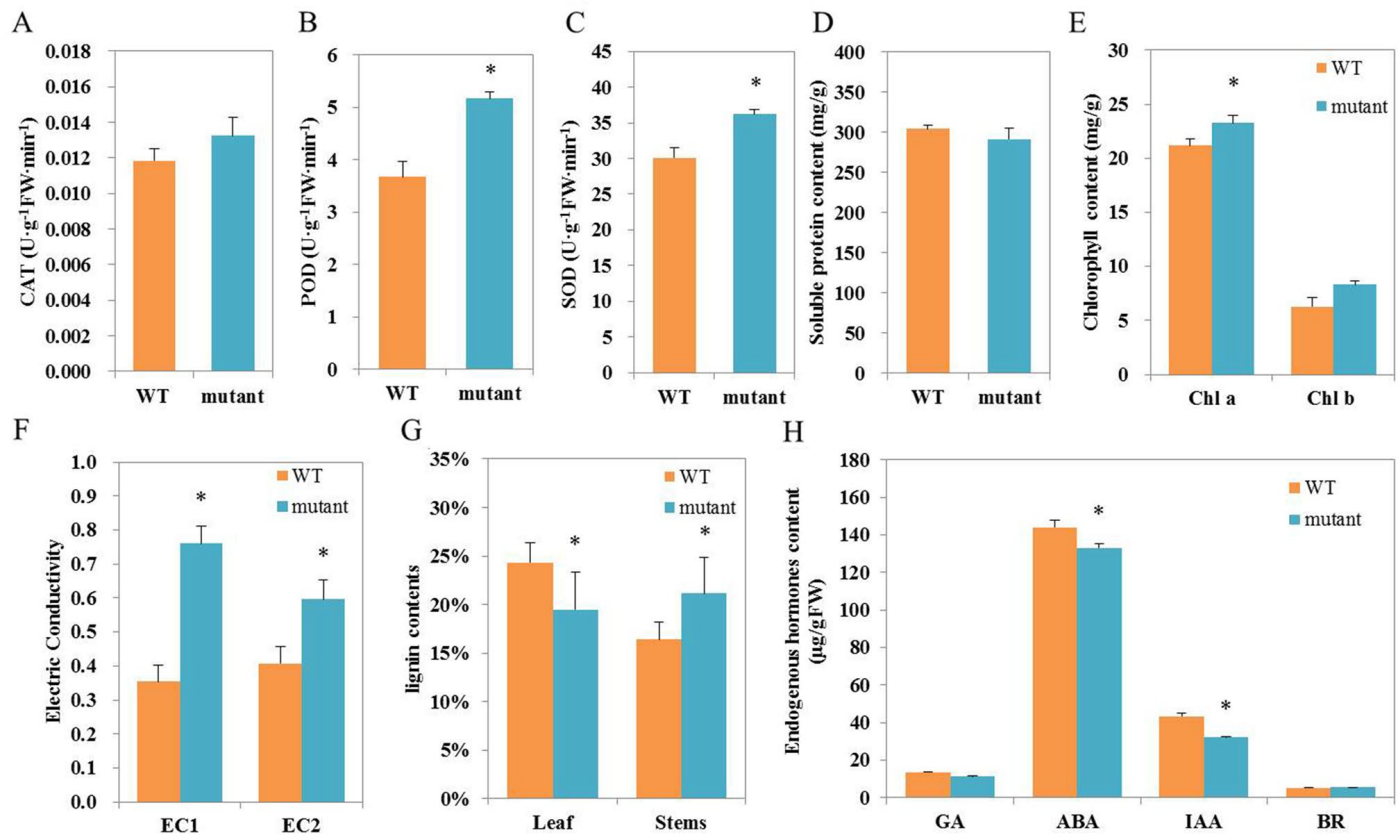
The physiological and biochemical differences between the dwarf mutant and WT plants of *Z*. *matrella*. (**A**) CAT. (B) POD. (**C**) SOD. (**D**) Soluble protein. (**E**) Chlorophyll a & b. (**F**) Electric conductivity. (**G**) Lignin content. (**H**) Endogenous hormones content. ‘*’ means the significant differences at *P*  < 0.05 by Duncan’s multiple range tests.

Plant physiological and biochemical variations are often used to characterize plant mutations^47^. In this study, we measured the activities of antioxidant enzymes (CAT, G-POD, and SOD), soluble protein, chlorophyll, electric conductivity, and lignin content in young leaves from both the WT and the mutant. The activities of G-POD and SOD significantly increased by 20% in the mutant (*p* = 0.05), while CAT showing slightly higher activity (Fig. 3A–C). The mutant and the WT plants had similar soluble protein contents (Fig. 3D). For the content of chlorophyll (Chl), both Chl a and b contents increased and the Chl b increased significantly in the mutant, and the ratios of Chl a to Chl b in the mutant and the WT were 2.83 and 3.36, respectively (Fig. 3E). The increased chlorophyll content was consistent with the greener leaves observed in the dwarf mutant. For relative conductivity, the conductivity of the mutant sample was twice as much as that of the WT (Fig. 3F). The containment of lignin in the mutant compared to that of in the WT plants reduced from 24 to 19% in leaves and increased from 16 to 21% in stem (Fig. 3G). These physiological changes provide additional evidence to verify that the dwarf mutant is different from the WT plants.

### Phytohormone content changes in the dwarf mutant

Several hormones can cause plant dwarfing^31^. The concentrations of GA and BR are often considered to be the main cause of dwarf plants^48^. To better understand the involvement of phytohormone in dwarf mutant, we measured the contents of four major endogenous hormones in leaves of the mutant and the WT plants. The contents of GA_3_ and BR in the mutant did not showed statistic differences from the WT plants, suggesting that they may not play a major role in leading to the dwarf mutant. In contrast, the contents of both ABA and IAA were decreased in the mutant. A significant IAA reduction of 26% was detected in the leaves of the mutant (Fig. 3H). The reduced IAA and ABA contents are likely responsible for the dwarfing in the mutant.

### Transcriptomic analysis of the mutant

A total of 50.1 Giga base (Gb) clean data, up to 27 million reads for each sample, were obtained (Supplemental Table 1). The clean reads (QC > 30) were mapped to the reference genome sequences of *Z. matrella*. The alignment efficiency of the clean reads to the reference genome was between 77.3 and 80.2%, indicating that the data was suitable for further analysis. By aligning to the reported reference genome (https://zoysia.kazusa.or.jp/), 1875 transcripts showed no hits and were mostly unannotated (Supplemental Table 2). These no hits may represent the genome specificity of the two genotypes that we used for RNA sequencing; meanwhile, further annotating these new genes can supplement the original genome annotation.

We first blasted the assembled sequences against the NR protein database. 36% of the sequences had e-values ranged from 1.0E^−5^ to 1.0 E^−50^, while 64% (39,409) of the sequences displayed E < 1.0E^−50^ (Supplementary Fig. 4A). Of the annotated reads, 26,440 showed 60–80% and 23,334 had 80–100% similarities to the known genes (Supplementary Fig. 4B). To study the sequence conservation of *Z. matrella* in other plant species, we analyzed the species distribution in the NR database. The closest species was *Setaria itallica*, with 24,521 genes (40.0%) matched. The second closest reference species was *Zea mays*, which showed 18.1% homology with *Z. matrella* (Supplementary Fig. 4C).

**Figure 4:**
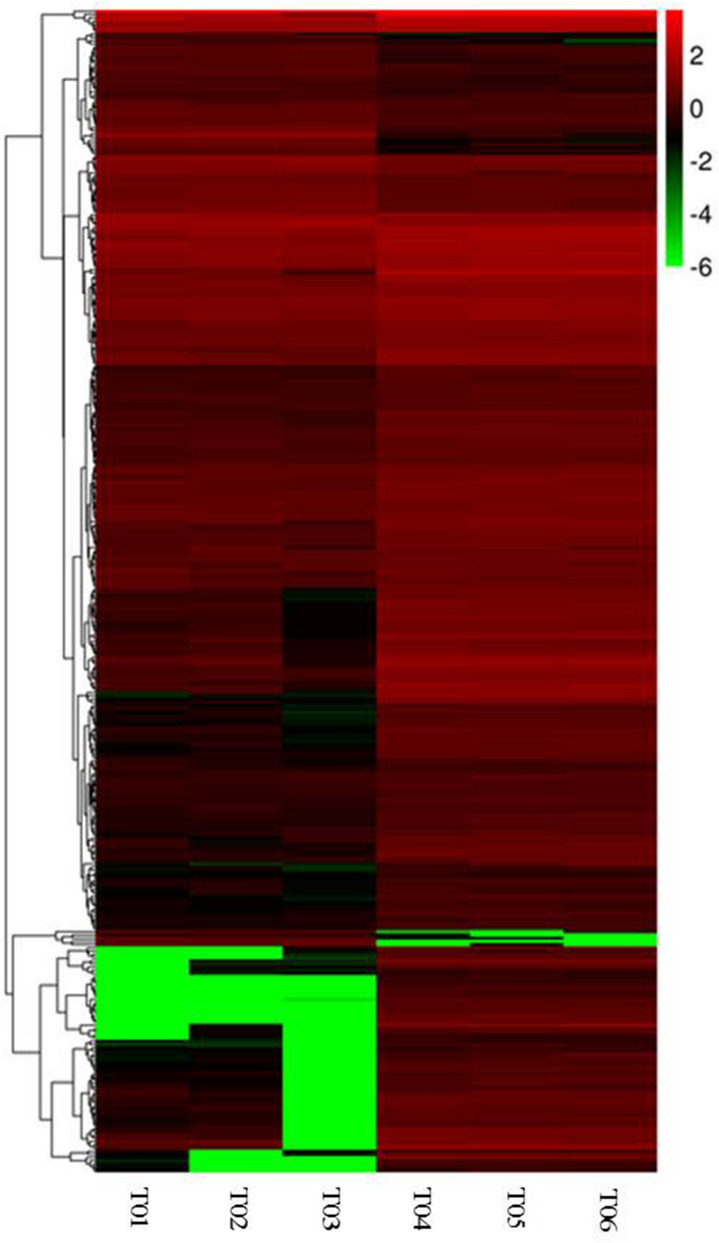
Heat map of DEGs expression between the dwarf mutant and WT. Each column represents a different sample (T01-03 dwarf mutant, T04-06 WT). Each line represents a different gene. Each color represents a different gene expression level with the scale bar on top-right corner.

The GO classification system was used to assess possible functions of the predicted genes. A total of 46,552 unigenes were assigned to 54 GO terms in three main GO ontologies. The top three frequently identified unigenes classified in the cellular component included ‘Cell part’ (32,677), ‘cell’ (32,475), and ‘organelle’ (28,696). ‘Binding’ (21,012), ‘catalytic activity’ (20,715), and ‘transporter activity’ (2769) were the top three GO terms in the molecular function. The top three GO terms in the biological processes were ‘Metabolic process’ (26,108), ‘cellular process’ (23,233), and ‘single-organism process’ (19,437) (Supplementary Fig. 5).

We then searched for all the unigenes in the COG database. 23,503 unigenes were identified, and they were classed into 25 functional groups (Supplementary Fig. 6). The largest group was ‘general function prediction only’ (18.8%), followed by ‘translation’ (9.7%) and ‘replication, recombination and repair’ (8.3%). The nuclear structures’ (0.01%), ‘cell motility’ (0.04%), and ‘chromatin structure and dynamics’ (0.8%) accounted for the least amounts.

The FPKM method was used to calculate the expression levels of genes to identify significant changes in gene expression in the mutant (Fig. 4 and Supplementary Fig. 6). A total of 360 DEGs including 62 up-regulated unigenes and 298 down-regulated ones, were identified in the comparison between the mutant and the WT leaves (Supplemental Table 3).

**Figure 5:**
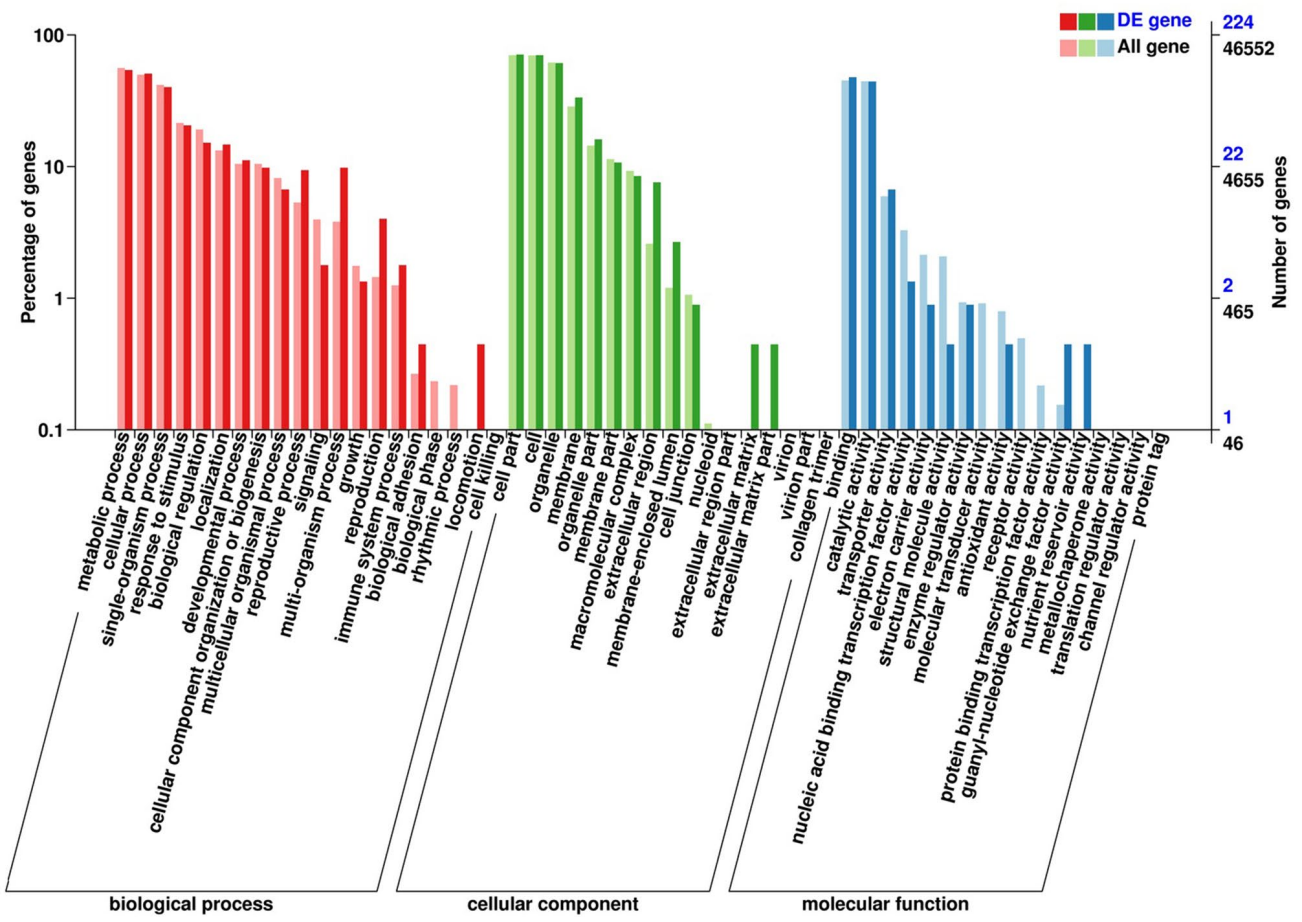
GO classification of the DEGs.

To reveal the function of the 360 DEGs, we assigned the DEGs to the GO terms. A total of 224 unigenes were assigned for 39 GO terms. The percentage of DEGs genes in some GO terms is significantly different from that of all genes, such as ‘reproductive process’, ‘signaling’, ‘multi-organism process’, ‘reproduction’, ‘extracellular region’, ‘membrane-enclosed lumen’, ‘electron carrier activity’, and ‘structural molecule activity’, suggesting that these DEGs may have involved in the dwarf mutant (Fig. 5).

**Figure 6:**
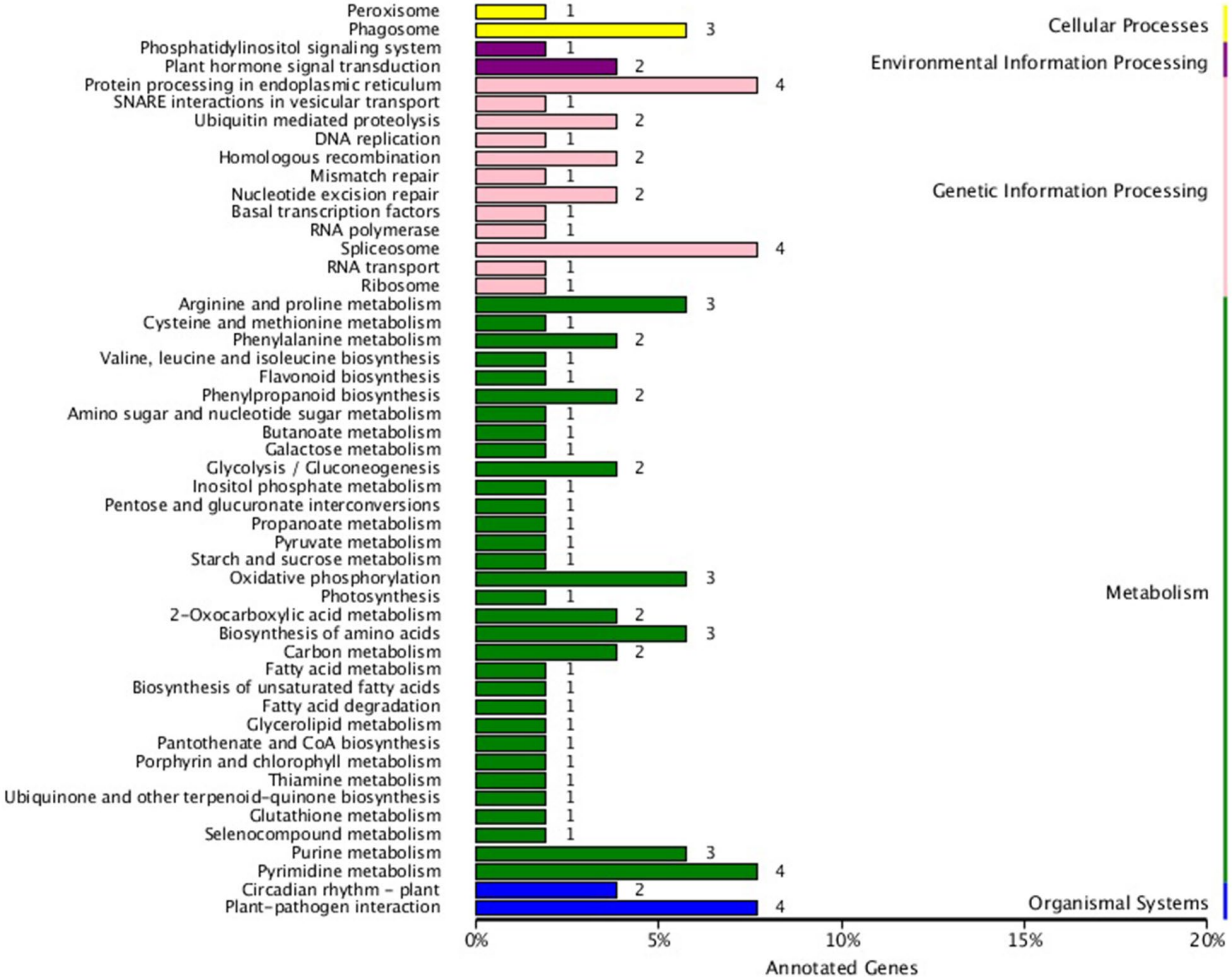
KEGG classification of DEGs. The number labeled on the column represents the number of DEGs identified in biochemical and signal transduction pathways.

KEGG pathway enrichment analysis was performed to identify the DEGs in biochemical pathways and signal transduction pathways. The main pathways identified were: ‘spliceosome’, ‘plant-pathogen interaction’, ‘protein processing in endoplasmic reticulum’, ‘pyrimidine metabolism’, ‘purine metabolism’, ‘phagosome’, ‘oxidative phosphorylation’, ‘biosynthesis of amino acids’, and ‘arginine and proline metabolism’, which contained the highest number of DEGs (Fig. 6). Among these genes, 22 DEGs are dwarf-related genes (Table 2), including IAA, BR, GA, ABA, cell wall, cytoskeleton and other dwarf-related pathway.

**Table 2:** DEGs detected in the comparison of the dwarf mutant and the WT leaves.

	Unigene ID	FDR	log_2_FC	NR_annotation
IAA pathway	Zmw_sc03690.1.g00070.1.sm.mk	3.85E−29	− 3.96427	Cytochrome P450; indole-2-monooxygenase
	Zmw_sc03749.1.g00050.1.am.mk	3.23E−50	− 4.47	Peptidyl-prolyl cis–trans isomerase 1 (Pin1)
	Zmw_sc03749.1.g00010.1.am.mk	1.28E−43	− 5.18	Pin1
	Zmw_sc01714.1.g00150.1.am.mk	7.57E−03	1.29	Calcium-binding protein CML12-like
BR pathway	Zmw_sc00216.1.g00070.1.am.mkhc	1.16E−03	− 1.03	TCP-1
GA pathway	Zmw_sc00373.1.g00120.1.sm.mkhc	8.86E−27	− 3.24	Disease resistance protein RGA2
ABA pathway	Zmw_sc01964.1.g00030.1.am.mk	1.87E−31	− 4.48	Cytochrome P450; abscisic acid 8′ -hydroxylase 2-like
Cell wall-cytoskeleton	Zmw_sc00161.1.g00260.1.sm.mkhc	7.29E−03	− 1.16	cellulose synthase1
	Zmw_sc00176.1.g00570.1.am.mk	3.71E−15	− 1.97	Expansin-B11
	Zmw_sc00343.1.g00200.1.am.mk	3.51E−06	− 1.29	Expansin-A4
	Zoysiamatrella_newGene_2340	3.98E−03	− 1.08	Expansin-B3-like
	Zmw_sc00491.1.g00050.1.sm.mk	2.50E−03	− 1.26	Expansin-A4
	Zoysiamatrella_newGene_2682	4.12E−10	− 1.52	Expansin-B6
	Zmw_sc02613.1.g00090.1.sm.mk	3.26E−06	− 1.35	Expansin-A4
	Zoysiamatrella_newGene_2400	4.18E−04	− 1.50	Expansin-B3
	Zoysiamatrella_newGene_1366	3.36E−07	− 1.28	Expansin-B6
	Zmw_sc08869.1.g00010.1.am.mk	1.48E−08	− 1.72	Expansin-B11
	Zoysiamatrella_newGene_1183	3.37E−12	− 1.67	Expansin-B3-like
	Zoysiamatrella_newGene_2399	1.86E−19	− 2.32	Expansin-B3-like
	Zoysiamatrella_newGene_2972	2.74E−13	− 1.81	Expansin-B2-like
Dwarf-related genes	Zmw_sc04963.1.g00040.1.sm.mkhc	4.87E−43	− 4.89	Transcription factor AP2
	Zmw_sc04225.1.g00030.1.am.mk	5.50E−21	− 3.85	Ocs element-binding factor 1-like

Log_2_FC = Log_2_Fold change (mutant/WT). FDR: False discovery rate.

### RT-qPCR analysis of dwarf-related DEGs

**Figure 7:**
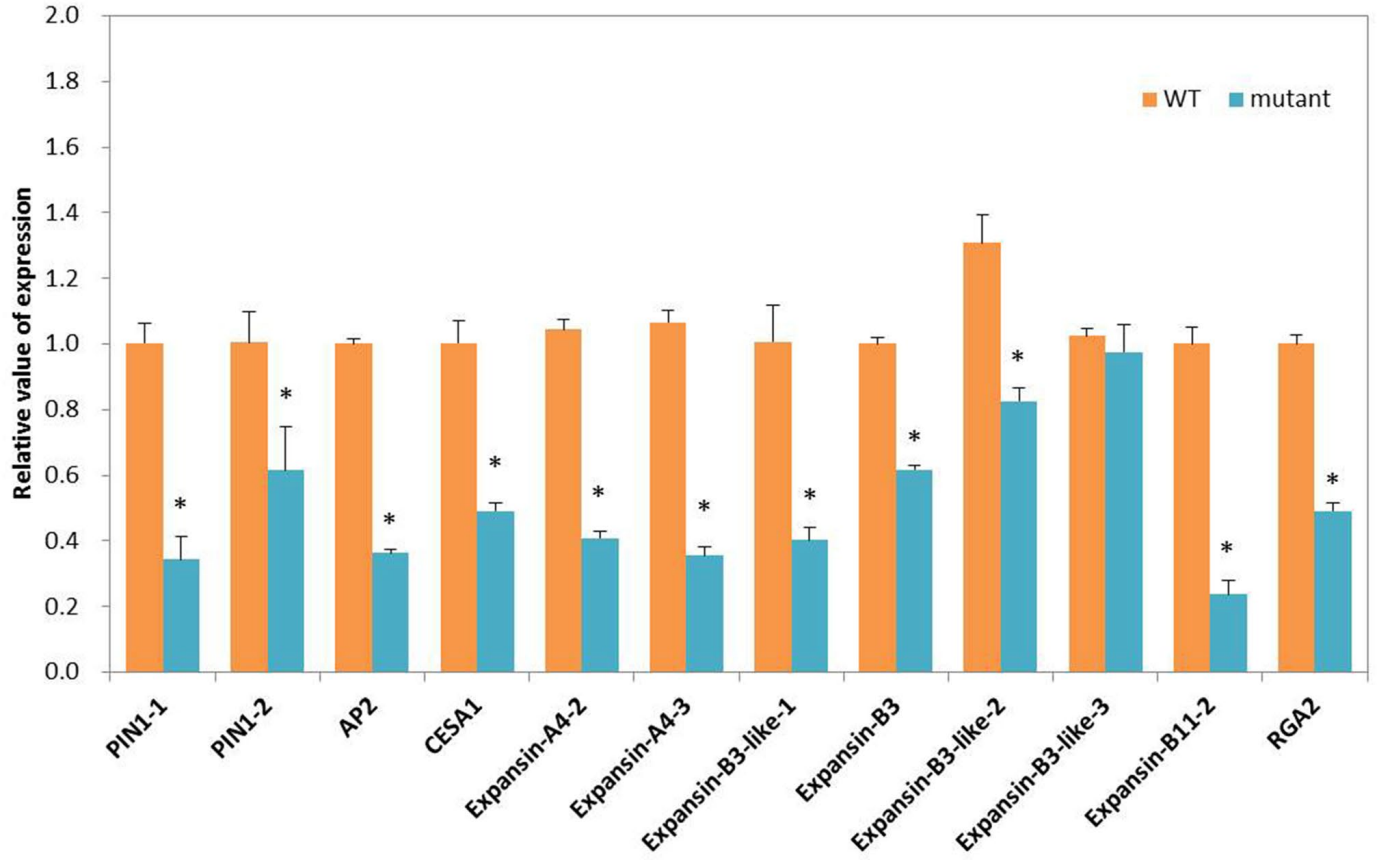
RT-qPCR analyses of 12 dwarf-related unigenes. ‘*’ means the significant differences at *P*  <  0.05 by Duncan’s multiple range tests.

RT-qPCR assays were performed for 12 dwarf-related unigenes from auxin pathway, BR pathway, gibberellin pathway, and cytoskeleton. The RT-qPCR results were consistent with those from RNA-sequencing analysis, although the exact fold change varied between the two techniques (Fig. 7). The results validated the DEGs identified in the RNA-sequencing analyses.

## Discussion

### The dwarf mutant is phenotypically stable

A stable dwarf mutant is of importance for dwarf plant breeding in *Z. matrella*. After three years’ propagation and observation, the mutant remained its dwarf trait, suggesting that this dwarf mutant is stable at least during clonal propagation. Since the mutant barely produced any seeds, we could not exam the next generation seedlings to determine whether genetic or epigenetic change(s) was responsible for the mutant.

Bulliform cells form a special structure of the leaf epidermis of gramineous plants, which is one of the reference indicators for drought tolerance^49^. Both the dwarf mutant and WT had similar bulliform cells at the veins (Supplementary Fig. 1), suggesting the mutant is likely not defective in drought resistance. Chl a/b ratio is an important indicator of plant shade tolerance^50^. The decreased Chl a/b ratio in the mutant not only makes the greener leaves of the dwarf mutant more attractive than the WT leaves but also indicates that shade tolerance of the mutant may have been changed and likely enhanced. As *Z. matrella* is asexually propagated into the lawn through stolon, we believe that the ^60^Co-γ-induced dwarf mutant is an invaluable material for research and turf industrials due to its slow growth for a potential to reduce the cost for mowing, attractive greener leaves, and a high potential of shade tolerance^51,52^.

### Antioxidant enzymes activity and other dwarf-related physiology

Of physiological and biochemical characteristics, the dwarf mutant exhibited differences, particularly in the activity of antioxidant enzymes (POD and SOD) (Fig. 3B,C), from the WT grass. Some of these differences were well correlated with the phenotypic changes in the mutant. For example, the activity of POD is often negatively correlated with stem elongation because of its impact on distribution of IAA in plants^53^. Therefore, there was no surprise that the increased POD was likely responsible, at least in part, for the dwarf mutant by enhancing oxidation of endogenous IAA decomposition. It was also possible that the accelerated formation of the xylem in the dwarf mutant caused an increase of electrolyte content in the leaves of the mutant, which is reflected by variation of relative conductivity.

### The DEGs of plant hormones in the dwarf mutant

Plant hormones are multifunctional in plant morphogenesis, growth, and metabolism^54^. Studies have shown that plant dwarfing mutations are closely related to GA, BRs, or IAA^55^. In rice and wheat, GA is one of the key factors that contribute to plant height^56–58^. Many dwarf mutants identified were related to BR biosynthetic and signaling pathways^59–61^. IAA is also a major determinant of plant growth by activating cell elongation^62^.

IAA regulates plant growth mainly through biosynthesis and transportation^63,64^. The biosynthesis of IAA is composed of two pathways: the tryptophan (Trp)-independent and Trp-dependent pathways^65^. Cytochrome P450s catalyze the first step of tryptophan-dependent indole-3-acetic acid biosynthesis. The double mutant cyp79b2cyp79b3 (cytochrome P450s were silenced) showed that the hypocotyls of seedlings were shortened, the plants were dwarfing, and the content of auxin was reduced^66^. PIN-FORMED (PIN) proteins are considered to be auxin efflux carriers, which play an important regulatory role in the polar transport of auxin^67,68^. The abnormal expression of key genes for auxin transport can cause plant dwarfism. Several DEGs related to auxin were down-regulated in the dwarf mutant lines of wheat induced by γ-ray irradiation, such as IAA synthesis and transport related genes^69^. In this study, the decreased IAA content and the IAA-related DEGs revealed that the dwarf mutant of *Z. matrella* was closely related to the IAA pathway. Biochemically, IAA synthesis in the dwarf mutant might have been reduced by both the decreased expression of the cytochrome P450 and the increased POD. The down-regulation of the cytochrome P450 indicated a reduction in IAA biosynthesis in the tryptophan-dependent pathway; and the reduction of auxin efflux carriers Pin1 decreased the IAA transportation as well.

ABA plays pleiotropic physiological roles in growth and exhibits potential relationship with plant dwarfing^68^. Cytochrome P450 enzymes are involved in degradation of ABA through 8ʹ-hydroxylase^70^. In this study, the down-regulated cytochrome P450 expression in ABA pathway could result in ABA accumulation due to a decreased ABA degradation, leading to the dwarf phenotype in the mutant.

The BR biosynthesis and signal transduction pathway were only recently clarified^71,72^. BR can control plant growth through cell elongation^73^. T-complex protein 1 (TCP1) regulates the synthesis of BR by regulating the expression of the *dwarf4* gene. The loss of the TCP1 gene function will lead to dwarf *Arabidopsis* plants and shorter hypocotyls that can be recovered by exogenous BR^74^. In this study, although the content of endogenous BR in the dwarf mutant did not show a significant change, a DEG annotated to *TCP1* in the BR pathway is down-regulated. Therefore, it was possible that BR was involved in the formation of the dwarf mutant.

### Cell wall and plant dwarfism

Cell wall is a three-dimensional network composed of polysaccharides, proteins, and other components formed by the intercrossing of cellulose and pectin^75^. The degree of cell wall extension can affect plant height. A mutation in the cellulose synthesis gene can also cause dwarfism^75^. The most widely-studied cellulose biosynthetic enzymes are Cellulose Synthase (CESA). In *Arabidopsis*, CESA1, CESA3, and CESA6 are essential for primary wall synthesis, preferentially expressed in swollen tissues, and their mutants exhibit an extremely dwarfed phenotype or lethal at seedling stage^76^. Similarly, in this study, the down-regulated *CESA1* observed provides additional evidence to verify the dwarf mutant at gene expression levels.

Expansin is a family of nonenzymatic proteins in the plant cell wall that are primarily involved in cell growth, elongation, and several cell wall modifications^77^. Increased expansin relaxes the cell wall tension and enables a higher degree of cell wall extension for taller plants. For example, the overexpression of OsEXP4 (an expansin gene) in transgenic rice resulted in taller plants by 12%^78^. In this study, thirteen down-regulated DEGs of expansin in the dwarf mutant suggested a potential of a reduced expansin accumulation and a reduced plant height. Previous studies have shown that genes encoding for expansin are positively related to lignin accumulation in a variety of plants^79,80^. Thus, lignin as the main component of the secondary cell wall in vascular plants can also reflect plant dwarfing indirectly^81^. In this study, higher lignin content in stem was detected in the dwarf mutant of *Z*. *metralla*, which is consistent with the findings of similar research in rice^82^. However, it is very interesting that in leaves approximately 20% reduction of acetyl bromide lignin levels was found in dwarf mutant leaves.

## Conclusions

A dwarf mutant of *Z. matrella* induced by gamma radiation was identified from callus regeneration. The significance of the transcriptome data, as well as the morphological and physiological characteristics, suggests that IAA transportation and expansin likely contribute to the major differences in the dwarfism of *Z. matrella*. This dwarf mutant provides a unique material to study plant growth and dwarf mechanism in turf grasses.

## Materials and method

### Plant material

In our previous study^1^, different doses of ^60^Co γ irradiation (0, 5, 10, 20, 40, 80, 100, 150, 200, 250, and 300 Gy) were used to study the impact of radiation exposure on callus growth and regeneration. The induced plants from the survival calluses after ^60^Co γ irradiation were transferred to a greenhouse to select desirable mutants. One dwarf line obtained under 100 Gy irradiation was selected according to leaf size, internode length, and leaf color. The mutant was transplanted, expanded, and pruned to observe its genetic stability. The regenerated plants from the calluses without irradiation were used as a wild type (WT) control.

### Morphological evaluation in the greenhouse

After 3 years in vitro propagation, dwarf mutants and WT plants spread through stolon in square basket container in the greenhouse. The plants were watered and fertilized as needed. Five phenotypic traits, including blade length, blade width, stolon internode length, stolon thickness, and plant height, were measured in mature plants in 2018. Each measurement was performed on 20 plants randomly^83^.

### Determination of enzyme activities and soluble protein

From each mutant and WT plant, three samples of fresh mature leaves were harvested from the greenhouse grown plants. The activity of the antioxidant enzyme was determined using a spectrophotometer, as previously described^1^. For each sample, 0.3 g of fresh leaves were ground at 4 °C in a pre-chilled mortar and pestle in 3 ml of 50 mM phosphate buffer (pH 7.8, 0.2 mM EDTA, 1% PVP) and centrifuged at 12,000 rpm for 20 min at 4 °C. The supernatant was recovered for determination of catalase (CAT), guaiacol peroxidase (G-POD), superoxide dismutase (SOD) and soluble protein. 50 μl of the supernatant in a 3 ml reaction solution, containing 25 mM phosphate buffer (pH 7.0) and 225 mM H_2_O_2_, was used to determine of CAT activity, the variation in absorbance of H_2_O_2_ (extinction coefficient 39.4 mM/cm) within 1 min at 240 nm was recorded. One unit of CAT activity was defined as the amount of enzyme required when 1 μmol H_2_O_2_ degraded into water per minute^84^. The G-POD activity was determined by the increase of absorbance at 470 nm, due to the oxidation of guaiacol, using 100 μl of the supernatant in a 3 ml reaction solution, containing 25 mM phosphate buffer (pH 7.0), 20 mM guaiacol, and 20 mM H_2_O_2_^85^. G-POD activity was calculated in terms of optical density of guaiacol oxidized min^−1^ g^−1^ fresh weight. The SOD activity was determined using a modified version of the method presented in Giannoplitis and Ries^86^, incubating 100 μl of the supernatant in 3 ml reaction solution, containing 50 mM phosphate buffer (pH 7.8, 0.1 mM EDTA), 130 mM methionine, 700 μM Nitrotetrazolium blue chloride (NBT), 13 μM riboflavin for 1 h at room temperature under 20 μmol m^−2^ s^−1^ fluorescent light. The absorbance was measured at 560 nm. One unit of SOD activity is defined as the amount of enzyme required to inhibit the photoreduction of NBT by 50%. The content of soluble protein was determined using coomassie brilliant blue G-250^87^. In each sample, 15 μl of supernatant was homogenized in 3 ml coomassie brilliant blue G-250 and absorbance was measured at 595 nm after 2 min. At the same time, the standard curve was made from bovine serum albumin. According to the standard curve and the OD_595_ value of the sample, the corresponding protein content (mg/g fresh weight) was calculated. Each determination was repeated three times.

### Determination of chlorophyll and relative conductivity

A modified version of the method describe by Tait and Hik was used to measure chlorophyll concentration^88^. Briefly, a total of 0.1 g of fresh leaves sliced into 5 mm pieces were soaked in 8 ml of DMSO for 72 h in dark at 25 °C. Chlorophyll extract was transferred to a cuvette and absorbances at 649 and 665 nm (Abs649 and Abs665) were measured by a spectrophotometer (UV-2550, SHIMADZU, Tokyo, Japan) and chlorophyll content was calculated using the equations: Chl a = 12.19 A_665_ − 3.45 A_649_ and Chl b = 21.99 A_649_ − 5.32A_665_. To determine relative conductivity, 0.1 g of fresh leaves sliced into pieces (< 5 mm) were soaked in 10 ml of deionized water for 2 h in a 32 ℃ water bath. Initial electrical conductivity of the medium (R1) was measured using an electric conductivity meter (DDB-6220, Shanghai LIDA Instrument Factory, China), and the final electrical conductivity (R2) was measured after the extract was autoclaved at 121 °C for 20 min and cooled to room temperature. Ion leakage was calculated as the ratio of R1/R2^89,90^.

### Determination of endogenous hormones

In total, 0.3 g of fresh leaves per sample were homogenized in 3 ml of extraction solution (80% methanol containing 1 mM of 2,6-Di-tert-Butyl-4-Methylphenol) using a mortar and pestle at 4 °C. The homogenate was then transferred to a 10 ml test tube, and this procedure was repeated twice. The mixture was inoculated for 4 h at 4 °C and was then centrifuged at 3500*g* for 8 min. The supernatant was collected and the residue was washed with 1 ml extraction solution. The supernatant was purified using a C-18 solid-phase extraction column, dried with methanol through nitrogen, then diluted with 2 ml sample diluent (PBS, containing 1% gelatin and 1% Tween-20). The contents of four endogenous hormones (i.e., IAA, GA3, ABA, and BR) were detected using Antibody-Based Enzyme-Linked Immunosorbent Assay, as reported by Deng et al.^91^. Absorbance was read at 492 nm on the microplate reader and the data were analyzed using ANOVA and Duncan with SPSS.

### Lignin contents

The acetyl bromide method was used to determinate lignin content^92^. 20 mg of dried leaves and stolon collected separately were reacted with 2 ml of 25% (v/v) acetyl bromide in acetic acid, sealed with Teflon lined caps heated at 70 °C in a water bath, and put in a shaking incubator for 30 min. Afterwards, 200 μl of the digesting mixture was transferred to a 10 ml centrifuge tube containing 0.45 ml of 2 M NaOH and 2.5 ml of acetic acid, 0.05 ml of 7.5 M hydroxylamine, as well as 5 ml acetic acid. The absorbance of the supernatant was measured at 280 nm after centrifuging at 3000*g* for 7 min. Lignin contents were calculated according to the standard curve. The total content of lignin is finally expressed by dividing the weight of lignin by the weight of the dried sample (%).

### Total RNA extraction and library construction for analysis

Total RNA was extracted from leaf tissues of the WT and dwarf mutant using the Trizol Reagent, following the instruction manual (Life Technologies, California, USA). All RNA samples were treated using DNase. RNA integrity was confirmed using a NanoDrop ND-1000 spectrophotometer (Thermo Scientific, Wilmington, DE, USA) and an Agilent 2100 Bioanalyzer (Agilent Technologies, Santa Clara, CA, USA). Samples with RIN ≥ 6.5 were used for RNA sequencing. A mixed cDNA library with different labels for the dwarf mutant and WT plants, respectively, was prepared according to the manufacturer’s instructions of NEBNext Ultra RNA Library Prep Kit for Illumina (NEB, E7530) and NEBNext Multiplex Oligos for Illumina (NEB, E7500).

### Sequencing, de novo assembly, and annotation

The cDNA libraries of the dwarf mutant and the WT were sequenced on a flow cell for 100 nt paired-end sequencing using an Illumina HiSeq 2500 sequencing platform with three biological replicates for each (Biomarker Technology Company, Beijing, China). All three replicates were run for de novo assembly of Illumina reads of the dwarf mutant and the WT. Clean reads were mapped to the genome of *Z. matrella* (https://zoysia.kazusa.or.jp/) using the TopHat2 Software^93^. The fragments per kilobase of exon per million fragments mapped (FPKM) values were used to estimate gene expression levels using the Cufflinks software^94^. BLAST was used to align genes to a series of protein databases, including NCBI non-redundant protein sequences (NR), NCBI non-redundant nucleotide sequences (Nt), Protein family (Pfam), Clusters of Orthologous Groups of proteins (KOG/COG), manually annotated and reviewed protein sequence database (Swiss-Prot), KO KEGG Ortholog database (KO), and Gene Ontology (GO) with a significance threshold of E ≤ 10^−5^^95,96^. All unigene sequences from *Z. matrella* have been deposited in the GenBank Sequence Read Archive (SRA) under accession number PRJNA666061 for SUB8197207.

### Identification and functional annotation of DEGs

DESeq^97^ and Q-value were used to evaluate differential gene expression between the dwarf mutant and the WT. After that, differences in gene abundance between samples were calculated based on the ratio of FPKM values. Significant DEGs were determined as expressed at a P < 0.01), and fold change (FC) > 2^98^.

### Quantitative reverse transcription PCR (RT-qPCR) verification

For qRT-PCR analyses, 1 μg total RNA per sample extracted from leaf tissues was used to synthesize cDNA with the PrimeScript RT Kit (TaKaRa, Japan). We subjected 12 dwarf-related unigenes to RT-qPCR analysis. The primers listed in supplementary Table 3 were designed using an online tool (https://www.genscript.com/tools/PCR-primers-designer). *ZmActin* was used as a housekeeping gene. Amplification was performed as follows: denaturation at 95 °C for 30 s, followed by 40 cycles of denaturation at 95 °C for 5 s, annealing at 60 °C for 15 s, and extension at 72 °C for 10 s. All reactions were performed in triplicates. Products were verified by melting curve analysis. The 2^−ΔΔCt^ method was used to calculate the relative expression levels of selected genes^99^.

## Supplementary information

Supplementary Figure Legends.

Supplementary Figure S1.

Supplementary Figure S2.

Supplementary Figure S3.

Supplementary Figure S4.

Supplementary Figure S5.

Supplementary Figure S6.

Supplementary Table S1.

Supplementary Table S2.

Supplementary Table S3.

Supplementary Table S4.
