# Supplementary material for: Analysis of a radiation-induced dwarf mutant of a warm-season turf grass reveals potential mechanisms involved in the dwarfing mutant
Source: Sci Rep. 2020 Nov 3;10:18913. doi: 10.1038/s41598-020-75421-x (PMC7609746; doi:10.1038/s41598-020-75421-x)
Supplement: Supplementary file 1 — Supplementary Figure Legends. [file 41598_2020_75421_MOESM1_ESM.docx]

**Supplementary Fig. 1 ­­­­The anatomical structure of dwarf mutant and WT mature leaves.** (A) Cross section of WT, ×40. (B) Cross section of the mutant, ×40. (C) Cross section of WT, ×200. (D) Cross section of the mutant, ×200.

**Supplementary Fig. 2 Morphological comparisons between the dwarf mutant and WT leaves according to ­the anatomical structure.** (A) Blade thickness. (B) Blade length. Label: ‘*’ on error bars mean the significant differences at *P < 0.05* by Duncan’s multiple range tests.

**Supplementary Fig. 3 A homology search was conducted by BLASTx against the NR database.** (A) E-value distribution of BLAST hits for matched unigene sequences. (B) Similarity distribution of top BLAST hits for each unigene. (C) Species distribution of the top BLAST hits.

**Supplementary Fig. 4 GO classification of the unigenes.**

**Supplementary Fig. 5 COG Function classification of assembled *Z. matrella* unigenes.**

**Supplementary Fig. 6 Identification of DEGs between the dwarf mutant and WT.** (A) Volcano plot: The x-axis shows the log of the fold change between both conditions; the y-axis shows the negative logarithm of the FDR. (B) MA plot: The x-axis represents the average expression (log scale) level between the two conditions, indicating the basal expression level. The y-axis is the fold change (log scale), which indicates the difference between the two.
