# Supplementary figures and images for: Analysis of a radiation-induced dwarf mutant of a warm-season turf grass reveals potential mechanisms involved in the dwarfing mutant
Source: Sci Rep. 2020 Nov 3;10:18913. doi: 10.1038/s41598-020-75421-x (PMC7609746; doi:10.1038/s41598-020-75421-x)

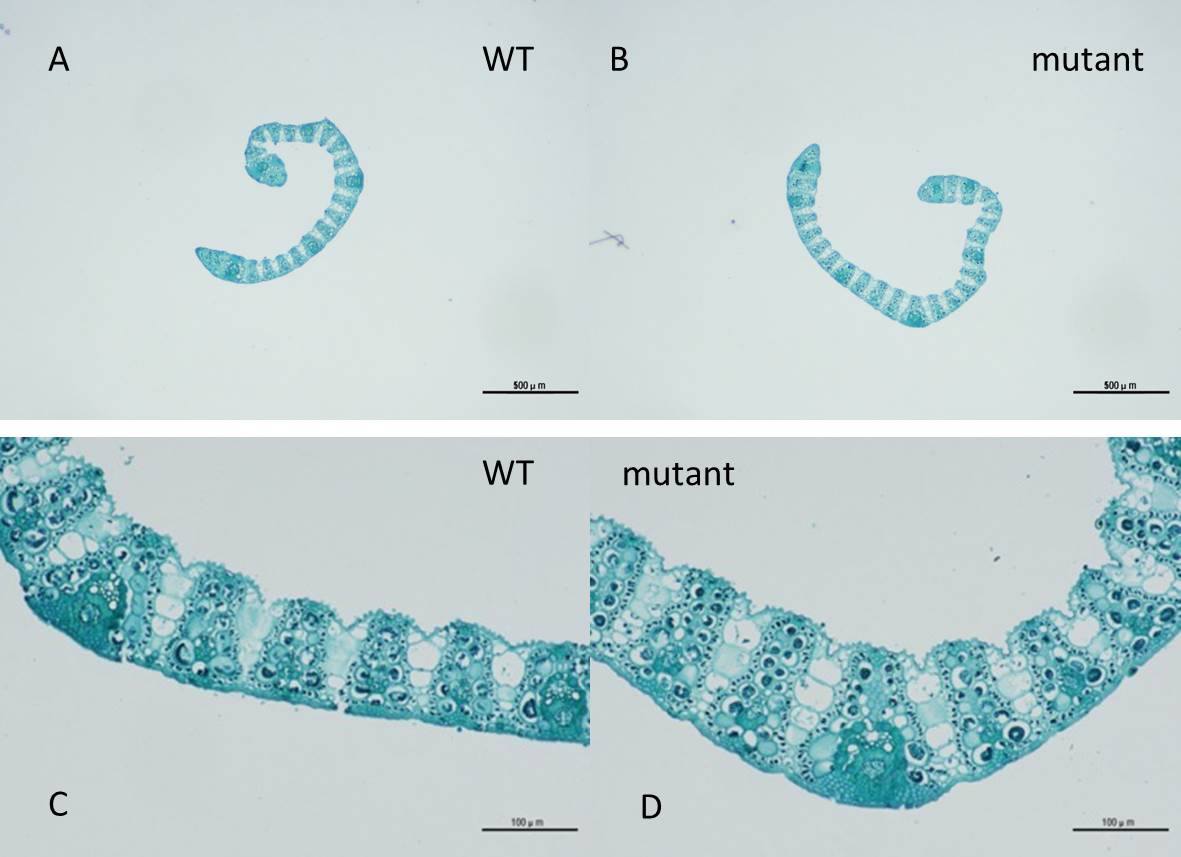

Supplement: Supplementary file 2 — Supplementary Figure S1. [file 41598_2020_75421_MOESM2_ESM.jpg]

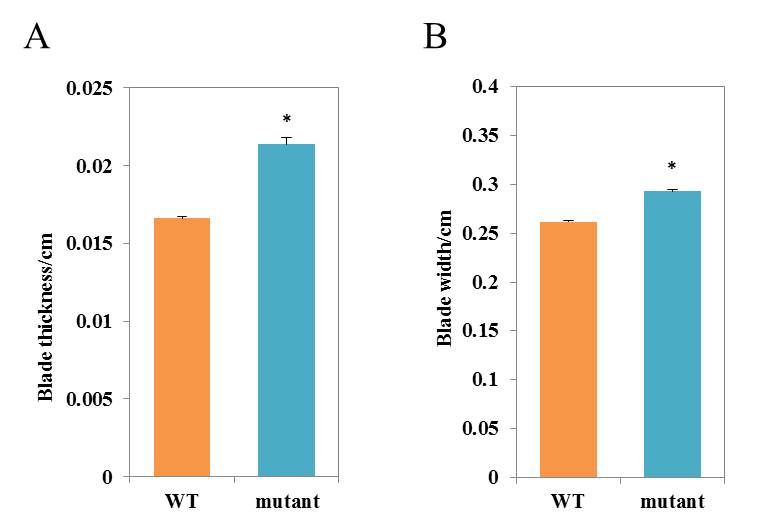

Supplement: Supplementary file 3 — Supplementary Figure S2. [file 41598_2020_75421_MOESM3_ESM.jpg]

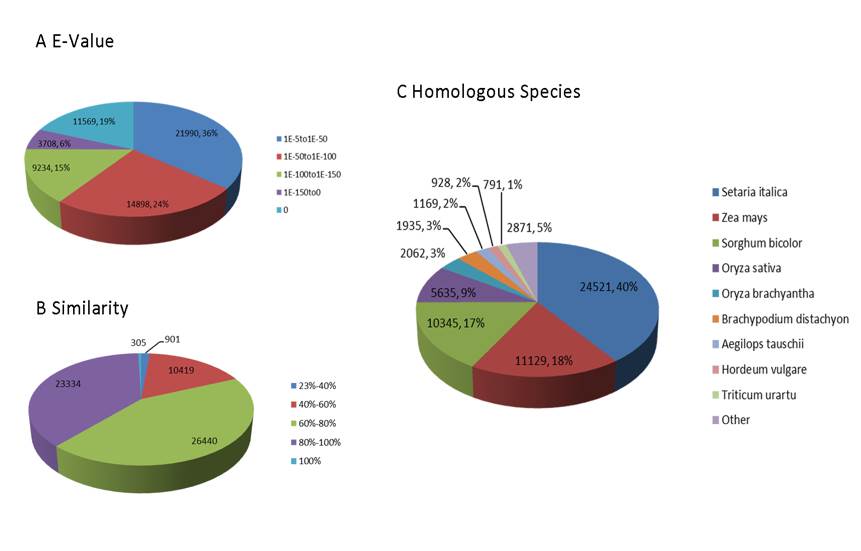

Supplement: Supplementary file 4 — Supplementary Figure S3. [file 41598_2020_75421_MOESM4_ESM.jpg]

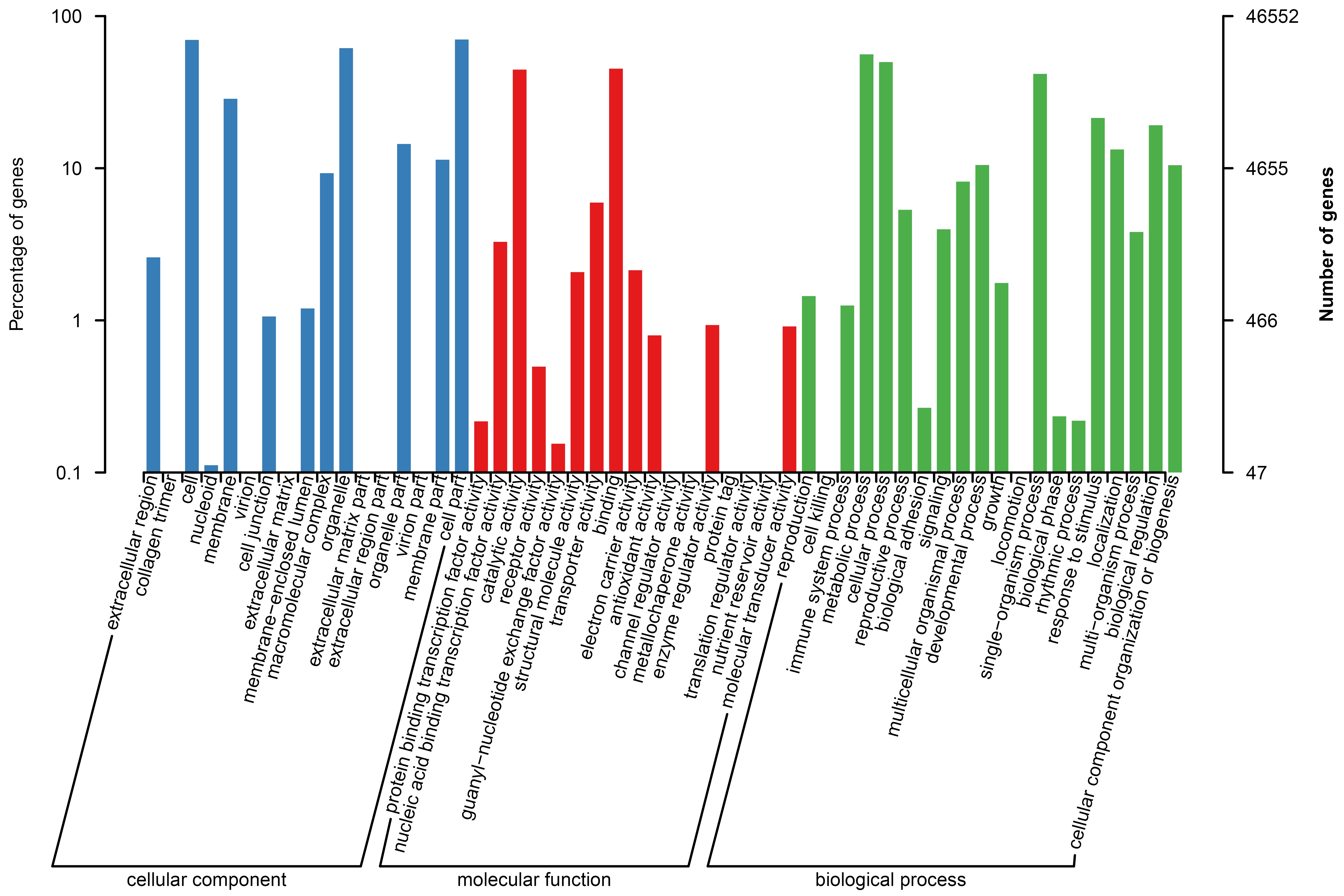

Supplement: Supplementary file 5 — Supplementary Figure S4. [file 41598_2020_75421_MOESM5_ESM.jpg]

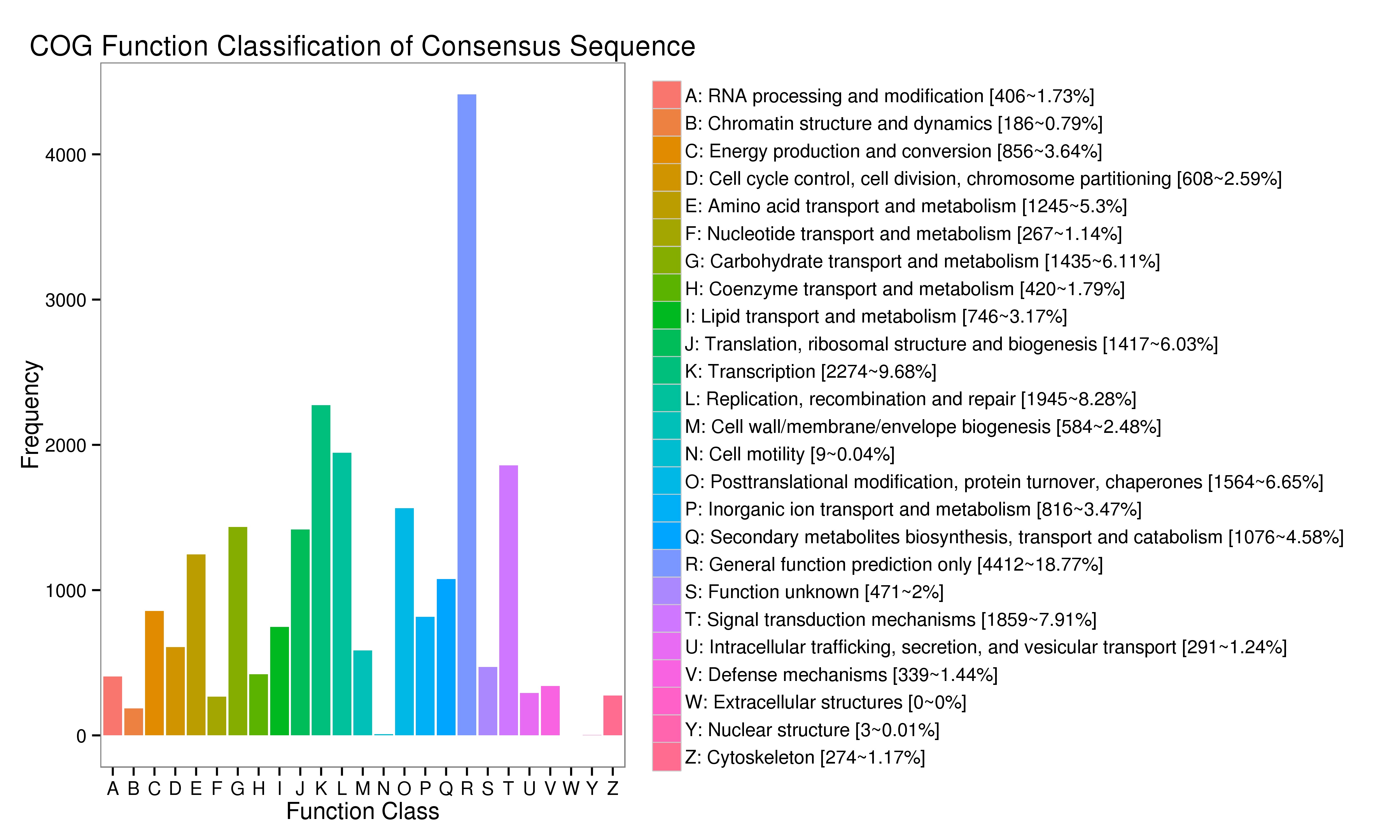

Supplement: Supplementary file 6 — Supplementary Figure S5. [file 41598_2020_75421_MOESM6_ESM.jpg]

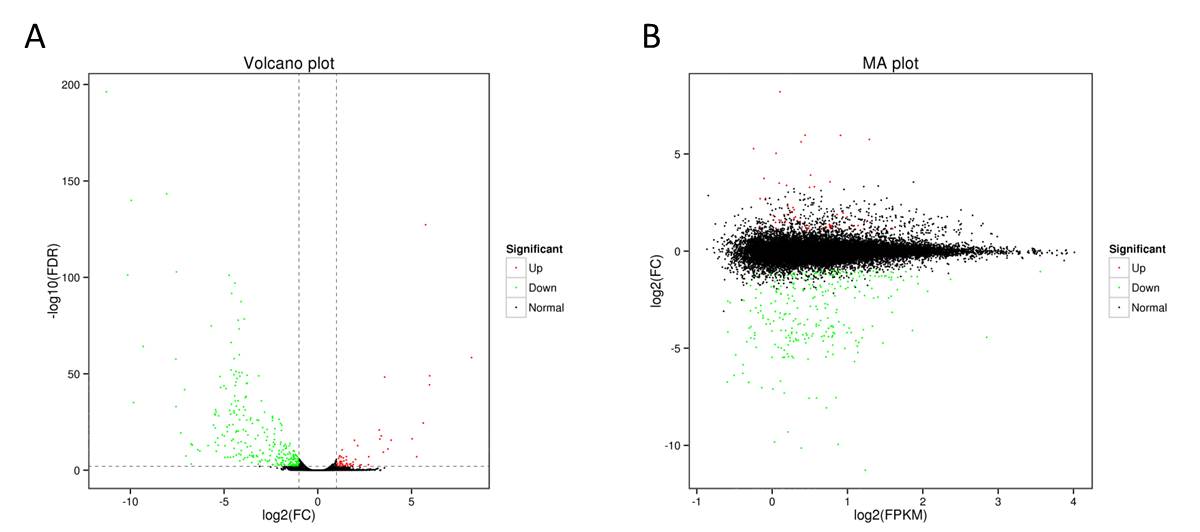

Supplement: Supplementary file 7 — Supplementary Figure S6. [file 41598_2020_75421_MOESM7_ESM.jpg]
